# Acute Coronary Syndrome in a Young Woman With Protein S Deficiency and Von Willebrand Disease Type 1: A Case of Mixed Hemostatic Disorders

**DOI:** 10.7759/cureus.87849

**Published:** 2025-07-13

**Authors:** Sofía Zapata Arceo, Ignacio de Jesus Garma Solis, Victor M Ayuso-Diaz, Angelica Moreno-Enriquez

**Affiliations:** 1 Internal Medicine, “Elvia Carrillo Puerto” High Specialty Regional Hospital, Institute for Social Security and Services for State Workers (ISSSTE), Mérida, MEX; 2 General Practice, Research and Education Division, Medical Care and Research, Yucatan, MEX; 3 General Practice, Genomic-Metabolic Unit, Marista University of Mérida, Mérida, MEX; 4 Molecular Biochemistry, Metabolic Genomic Unit, Marista University of Mérida, Mérida, MEX

**Keywords:** arterial thrombosis, bleeding diathesis, coagulation profile, inherited coagulation disorders, myocardial infarction in young adults, platelet dysfunction, postpartum hypercoagulability, protein s deficiency, thrombophilia, von willebrand disease type 1

## Abstract

Acute myocardial infarction (AMI) in young adults without conventional cardiovascular risk factors is rare and is often associated with non-atherosclerotic causes, including hereditary thrombophilias. Although protein S deficiency is classically associated with venous thrombosis, it has increasingly been implicated in arterial events. The postpartum period is a state of physiological hypercoagulability, which can reveal underlying prothrombotic conditions. Here, we present a case of a 31-year-old woman who developed septoapical AMI one week following elective cesarean section. Coronary angiography revealed thrombosis of the left coronary trunk and anterior descending artery, with no evidence of atherosclerotic lesions. She underwent percutaneous intervention with multiple stents and commenced anticoagulation therapy with acenocoumarol following the identification of partial protein S deficiency (56%).

During follow-up, she experienced spontaneous mucocutaneous bleeding, prompting further investigation. Prolonged activated partial thromboplastin time (aPTT) and reduced activity of factor VIII (20.2%), von Willebrand factor (23.2%), and von Willebrand factor antigen (31.3%), as well as platelet hypofunction on aggregometry, led to a diagnosis of type 1 von Willebrand disease being made. The anticoagulant regimen was switched to dabigatran. The bleeding disorder was likely unmasked by anticoagulation. The final diagnosis was established based on platelet hypofunction and coagulopathy findings, which also guided the therapeutic adjustment. The patient achieved a favorable clinical outcome, characterized by symptom resolution, an absence of new thrombotic or bleeding events, and partial biochemical recovery. This case highlights the importance of comprehensive hematological investigations in young patients presenting with atypical arterial thrombotic events.

## Introduction

Acute myocardial infarction (AMI) in young patients without traditional cardiovascular risk factors is uncommon and requires consideration of non-atherosclerotic etiologies. Among these, coagulation disorders, both hereditary and acquired, are particularly relevant. Thrombophilias, such as congenital protein S deficiency, have been reported as contributing factors, particularly when they occur alongside transient procoagulant states, such as the puerperium [1,2].

During pregnancy and the early postpartum period, the hemostatic balance shifts towards a hypercoagulable state. There are increases in fibrinogen and procoagulant factors (VII, VIII, X, and von Willebrand factor), alongside reductions in endogenous anticoagulants, such as free protein S and activated protein C. While these changes reduce the risk of postpartum hemorrhage, they may also reveal previously silent prothrombotic disorders [1,3].

Congenital protein S deficiency affects 0.03-0.13% of the general population. Diagnosis is often hindered by the overlap between normal and pathological ranges, and by the influence of physiological or pharmacological variables, including pregnancy and anticoagulant therapy, on assay results. Although it is traditionally associated with venous thromboembolism, its link with arterial events, particularly in young people, is becoming more widely recognized [4-6].

Von Willebrand disease (VWD), the most common inherited bleeding disorder, is characterized by a partial or complete deficiency of von Willebrand factor, typically presenting with mucocutaneous bleeding. However, it may remain clinically silent until triggered by surgery, trauma, or anticoagulation therapy. Type 1 VWD, which represents over 70% of cases, is characterized by quantitatively reduced but often borderline factor levels. This can lead to diagnostic uncertainty, particularly in patients receiving anticoagulants, since some coagulation assays may be altered or falsely normalized by the therapy [7,8].

In the event of a thrombotic episode, the presence of an underlying bleeding disorder, such as von Willebrand disease (VWD), poses diagnostic and therapeutic challenges. The coexistence of opposing hemostatic phenotypes (prothrombotic and hemorrhagic) requires careful interpretation of laboratory results and regular re-evaluation. In such cases, platelet aggregometry is crucial, particularly when platelet hypofunction is suspected or bleeding persists despite correction of coagulation factor levels. This approach was essential in the present case, in which a young postpartum patient presented with acute myocardial infarction (AMI) followed by delayed hemorrhagic manifestations that prompted re-evaluation of her hemostatic profile.

## Case presentation

A 31-year-old woman with a history of type I osteogenesis imperfecta and no prior chronic diseases, surgeries, or known allergies experienced sudden chest pain and dyspnea one week after an uncomplicated cesarean section. Upon presentation to the emergency department, an ECG revealed ST-segment elevation in the septoapical leads (V2-V5), and cardiac biomarkers were markedly elevated, consistent with ST-elevation myocardial infarction (STEMI). Transthoracic echocardiography revealed apical hypokinesis.

**Figure 1:**
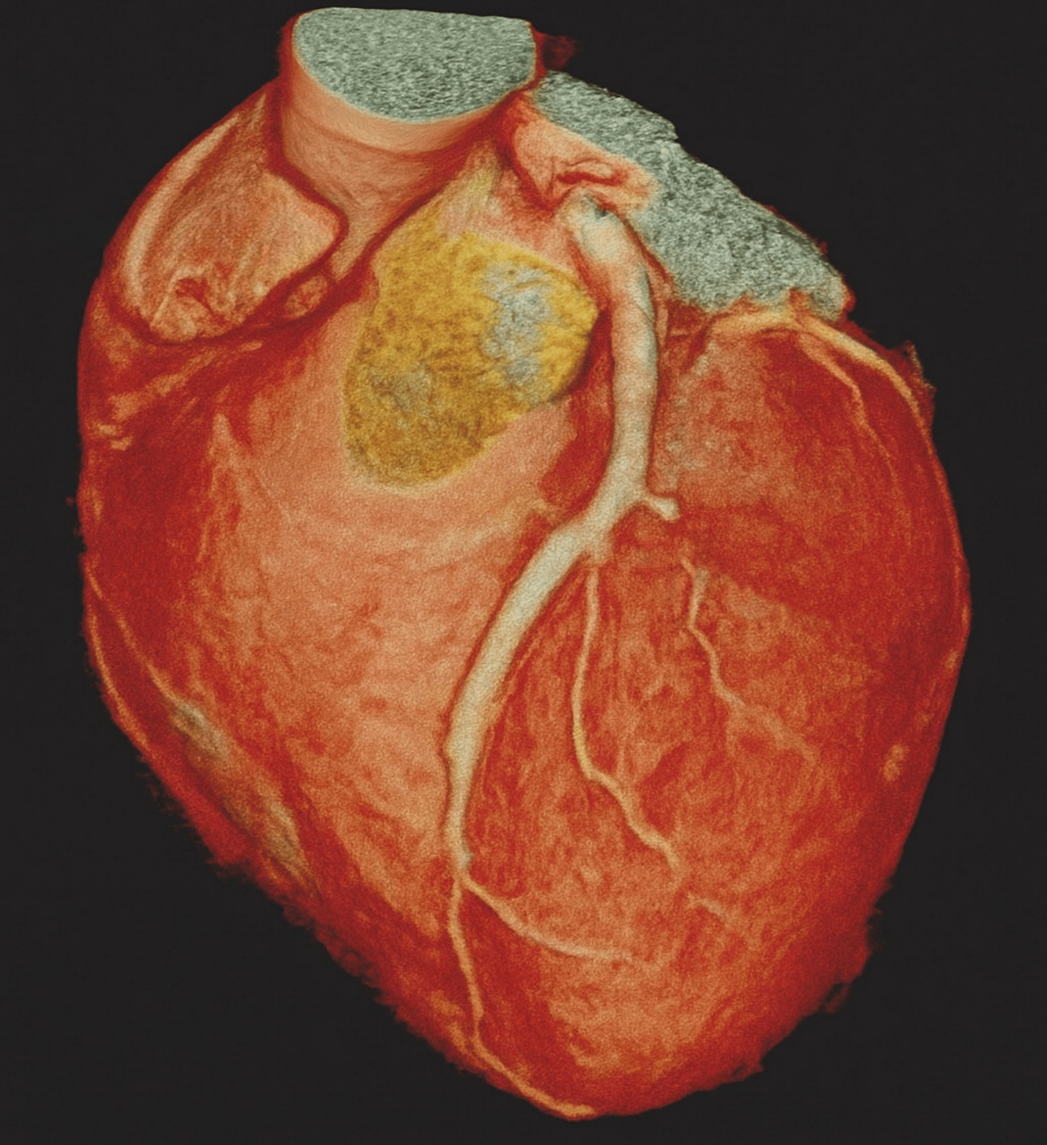
Three-dimensional reconstruction from coronary CT angiography. The image shows the trajectory of the left coronary trunk and anterior descending artery (LAD), with no evidence of atherosclerotic plaques or calcification. Although not a substitute for invasive coronary angiography, this reconstruction supports the thrombotic etiology in the context of hereditary thrombophilia in a young patient with no traditional cardiovascular risk factors.

Emergency coronary angiography revealed thrombotic occlusion of the left main trunk and proximal left anterior descending (LAD) artery, with no angiographic evidence of atherosclerotic plaque (Figure 1). Percutaneous coronary intervention (PCI) was performed, involving the placement of two drug-eluting stents and the administration of tirofiban (a glycoprotein IIb/IIIa inhibitor) and unfractionated heparin at a weight-adjusted dose (70 U/kg). Dual antiplatelet therapy (DAPT) was initiated with 100 mg of aspirin and 75 mg of clopidogrel daily.

A second PCI was performed 48 hours later due to persistent thrombus and 95% LAD occlusion, with a third stent being implanted. Intravascular imaging (IVUS or OCT) and thrombus aspiration were not performed due to resource constraints. The patient was discharged on acenocoumarol and DAPT, with a target international normalized ratio (INR) of 2.0-3.0.

During the subsequent 24 months, she remained free from thrombotic events. However, she developed spontaneous mucocutaneous bleeding, including purpura, gingivorrhagia, and polymenorrhea. Her INR remained within the therapeutic range (2.4) during these episodes, and she had not been exposed to any interacting medications (e.g., antibiotics). This prompted a re-evaluation of her hemostatic profile.

Coagulation studies revealed a prolonged activated partial thromboplastin time (aPTT), alongside normal PT and INR. Mixing studies corrected the aPTT, suggesting a factor deficiency. Further testing revealed reduced factor VIII activity (20.2%), von Willebrand factor activity (23.2%), and von Willebrand antigen (31.3%). Platelet aggregometry using adenosine diphosphate (ADP), adrenaline, and ristocetin revealed global hypofunction. These results supported a diagnosis of von Willebrand disease (type 1), which was complicated by underlying protein S deficiency (Table 1).

**Table 1:** Hemostatic profile during the diagnostic approach for thrombophilia and bleeding diathesis. Key results of the coagulation studies during the diagnostic evaluation of a patient with acute myocardial infarction in the absence of atherosclerosis, followed by spontaneous bleeding. The findings included protein S hypofunction, prolonged aPTT corrected by mixing study (suggesting factor deficiency), reduced coagulation factor levels, and global platelet hypofunction. These results supported the coexistence of hereditary thrombophilia with an underlying bleeding disorder of mixed origin. ADP: adenosine diphosphate; ADR: adrenaline; RIS: ristocetin; aPTT: activated partial thromboplastin time

Parameters	Patient result	Reference range
Protein S activity (%)	56	60-150
aPTT (s)	Prolonged	25-37
PT (s)	Normal	11-15
INR	Normal	0.9-1.2 (without anticoagulation)
Mixing study (aPTT)	Corrects	Corrects if deficiency present
Factor VIII activity (%)	20.2	50-150
von Willebrand factor activity (%)	23.2	50-150
von Willebrand antigen (%)	31.3	50-150
Platelet aggregation (ADP, ADR, RIS)	Decreased	≥70% response (normal)

Due to persistent bleeding despite therapeutic INR levels, acenocoumarol was replaced with 110 mg of dabigatran twice daily. Using a direct oral anticoagulant with a more predictable pharmacokinetic profile enabled safer anticoagulation. The patient experienced complete resolution of clinical bleeding, with no further hemorrhagic or thrombotic episodes. There was partial improvement in factor VIII and von Willebrand factor levels. Genetic or familial testing for von Willebrand disease was not performed. Osteogenesis imperfecta was considered incidental as there was no laboratory or clinical evidence of associated platelet or connective tissue dysfunction in this case.

Based on the sequential findings, a definitive diagnosis of type 1 von Willebrand disease superimposed on thrombophilia due to partial protein S deficiency was made. The patient underwent multidisciplinary follow-up for 24 months. During this period, bleeding episodes resolved completely, and there were no recurrences of thrombotic events. Hemostatic parameters progressively improved during follow-up, including normalization of aPTT, recovery of factor VIII (42%) and von Willebrand factor activity (48%), and improvement in protein S levels. This clinical and biochemical evolution is summarized in Table 2.

**Table 2:** Clinical, therapeutic, and laboratory evolution during 24-month follow-up. Longitudinal follow-up of a patient with a combined diagnosis of thrombophilia and type 1 von Willebrand disease, showing clinical stabilization, therapeutic adjustments, and progressive recovery of hemostatic parameters. The patient remained asymptomatic, with no recurrence of thrombotic or hemorrhagic events. ADP: adenosine diphosphate; ADR: adrenaline; RIS: ristocetin; aPTT: activated partial thromboplastin time; INR/PT: international normalized ratio

Parameters	Initial value	24-month value	Reference range
Protein S activity (%)	56	70	60-150
aPTT (s)	Prolonged	Normal	25-35
INR/PT (s)	Normal	Normal	0.9-1.2/11-15
Factor VIII activity (%)	20.20	42	50-150
von Willebrand factor activity (%)	23.20	48	50-150
von Willebrand antigen (%)	31.30	51	50-150
Platelet aggregation (ADP, ADR, RIS)	Decreased	Normal	≥70% response

## Discussion

Acute myocardial infarction (AMI) in young patients without conventional cardiovascular risk factors poses a significant diagnostic challenge and often requires the investigation of non-atherosclerotic causes. Hereditary thrombophilias, such as congenital protein S deficiency, are particularly relevant in such cases, especially when they occur alongside transient prothrombotic states, such as the postpartum period [9-11]. In this case, AMI occurred one week after cesarean section in a previously healthy young woman, in the context of physiological hypercoagulability and decreased protein S activity.

The patient presented with thrombotic occlusion of the left main coronary artery and the proximal left anterior descending artery (LAD), with no angiographic evidence of atherosclerosis (Figure 1). Vital signs at presentation were stable, and despite the extent of coronary occlusion, the patient did not develop cardiogenic shock. ST-segment elevation on an ECG and elevated cardiac biomarkers confirmed a diagnosis of STEMI.

Spontaneous coronary artery dissection (SCAD) was considered as a differential diagnosis. However, the absence of angiographic features suggestive of SCAD (such as multiple radiolucent lumens, long tubular narrowing, or spiral dissections), along with the presence of a large thrombus and favorable response to antithrombotic therapy, supported a thrombotic etiology. Intravascular imaging (IVUS or OCT) was not performed due to limited availability; this remains a recognized limitation of this report.

Although protein S deficiency is classically associated with venous thromboembolism, there is evidence to suggest an association with arterial events, including stroke and myocardial infarction, particularly in younger patients with transient prothrombotic triggers [4,12]. In this case, mucocutaneous bleeding, including purpura, gingivorrhagia, and polymenorrhea, prompted reassessment of hemostatic status. Functional studies revealed significant reductions in von Willebrand factor activity and antigen levels, as well as factor VIII deficiency and platelet hypofunction. This led to a diagnosis of type 1 von Willebrand disease (VWD) [8,13].

The coexistence of hereditary thrombophilia and a bleeding disorder, such as VWD, is rare and has rarely been reported in the literature. A recent review identified fewer than 10 published cases of such dual pathology, most of which involved venous thromboembolism rather than arterial thrombosis. The pathophysiological basis remains unclear, though independent inheritance patterns are possible, and there may be synergistic effects on endothelial dysfunction and platelet regulation. In our case, however, osteogenesis imperfecta was deemed incidental, with no evidence of altered platelet aggregation or connective tissue fragility contributing to the clinical picture.

From a diagnostic standpoint, this case highlights the importance of reconsidering initial diagnoses when new clinical features emerge and of using functional platelet testing when bleeding continues despite the standard correction of coagulation factors. The partial recovery observed in factor VIII and von Willebrand levels over 24 months may reflect the combined effects of the treatment response, laboratory variability, and the physiological fluctuations inherent in type 1 von Willebrand disease (VWD).

Regarding anticoagulation, acenocoumarol was replaced with dabigatran. The patient's stable renal function guided this decision, the requirement for predictable pharmacokinetics without international normalized ratio (INR) monitoring, and the lower bleeding risk associated with direct thrombin inhibitors compared to vitamin K antagonists. Among direct oral anticoagulants (DOACs), dabigatran was selected due to its favorable safety profile in patients at risk of bleeding and its reversibility.

Alternative differential diagnoses, such as antiphospholipid syndrome, vasculitides, hyperhomocysteinemia, and paraneoplastic prothrombotic states, were considered and systematically excluded by the rheumatology and hematology services. This highlights the importance of a multidisciplinary approach when dealing with atypical thrombotic events in young adults.

## Conclusions

Partial protein S deficiency should be considered when evaluating young patients with arterial thrombotic events and no conventional cardiovascular risk factors. This highlights the diagnostic complexity of overlapping thrombotic and bleeding disorders, including type 1 von Willebrand disease, as well as the necessity of tailoring anticoagulation therapy to the individual and guiding it with dynamic hemostatic monitoring.

The patient's 24-month follow-up was clinically uneventful, with complete resolution of bleeding symptoms, no recurrence of thrombotic events, and an improvement in coagulation parameters over time. This case further emphasizes the importance of re-evaluating initial diagnoses when new clinical manifestations emerge, such as delayed hemorrhagic symptoms, particularly in patients initially managed for isolated thrombophilia. A multidisciplinary approach enabled timely therapeutic adjustments and favorable long-term outcomes.
